# Surface ID: a geometry-aware system for protein molecular surface comparison

**DOI:** 10.1093/bioinformatics/btad196

**Published:** 2023-04-17

**Authors:** Saleh Riahi, Jae Hyeon Lee, Taylor Sorenson, Shuai Wei, Sven Jager, Reza Olfati-Saber, Yanfeng Zhou, Anna Park, Maria Wendt, Hervé Minoux, Yu Qiu

**Affiliations:** Large Molecule Research, Sanofi, Cambridge, MA 02141, United States; Data & Data Science, Sanofi, Cambridge, MA 02141, United States; Present address: Prescient Design, Genentech, Inc., South San Francisco, CA 94080, USA; Data & Data Science, Sanofi, Cambridge, MA 02141, United States; Large Molecule Research, Sanofi, Cambridge, MA 02141, United States; Present address: Bristol Myers Squibb, 100 Binney St, Cambridge, MA 02142, USA; R&D Digital Data & Computational Sciences, Sanofi, Industriepark Hoechst, Frankfurt am Main 65929, Germany; Data & Data Science, Sanofi, Cambridge, MA 02141, United States; Large Molecule Research, Sanofi, Cambridge, MA 02141, United States; Large Molecule Research, Sanofi, Cambridge, MA 02141, United States; Large Molecule Research, Sanofi, Cambridge, MA 02141, United States; Data & Data Science, Sanofi, Chilly-Mazarin 91380, France; Large Molecule Research, Sanofi, Cambridge, MA 02141, United States

## Abstract

**Motivation:**

A protein can be represented in several forms, including its 1D sequence, 3D atom coordinates, and molecular surface. A protein surface contains rich structural and chemical features directly related to the protein’s function such as its ability to interact with other molecules. While many methods have been developed for comparing the similarity of proteins using the sequence and structural representations, computational methods based on molecular surface representation are limited.

**Results:**

Here, we describe “Surface ID,” a geometric deep learning system for high-throughput surface comparison based on geometric and chemical features. Surface ID offers a novel grouping and alignment algorithm useful for clustering proteins by function, visualization, and *in silico* screening of potential binding partners to a target molecule. Our method demonstrates top performance in surface similarity assessment, indicating great potential for protein functional annotation, a major need in protein engineering and therapeutic design.

**Availability and implementation:**

Source code for the Surface ID model, trained weights, and inference script are available at https://github.com/Sanofi-Public/LMR-SurfaceID.

## 1 Introduction

Proteins are a class of macromolecules that perform a diverse range of functions in the cell. These functions are largely achieved by their 3D structures, which are in turn determined by their genetically encoded amino acid sequences. The relationship between a protein’s function and its structure or sequence has been long studied. Many methods have thus been developed to compare and classify proteins using overall sequence and structural similarities (Pearson and Lipman 1988; Altschul et al. 1990; Holm and Sander 1993; Ma and Wang 2014; Chatzou et al. 2016; Wang et al. 2018; Carpentier and Chomilier 2019). Although they have shown success, these types of methods have limitations as similarities in sequence and structure do not necessarily link to similarities in function. During evolution or in response to a selection pressure, different protein functions can be derived from the same structural scaffold. For example, antibodies can have very diverse functions although they possess similar overall sequences and structures (Chiu et al. 2019). In contrast, proteins with different structures can have similar functions. For instance, viral proteins can mimic the functions of cellular proteins to interfere with key cellular processes although the structures of viral and cellular proteins are very different (Alcami 2003). As demonstrated in these cases, protein function stems from the local structural elements, such as chemical properties and geometric shape instead of overall sequence and structure scaffold. Therefore, a method that can accurately describe and compare such function-related local structural features is greatly needed.

A protein’s molecular surface is a compact smooth surface composed of atoms on its boundary, displaying patterns of both chemical and geometric features. Compared with sequence and structure, a protein’s surface is more directly related to biomolecular interaction and function (Tseng and Li 2012). Despite the importance of surfaces, molecular surface comparison methods have not been extensively studied compared with sequence- and structure-based methods. One reason is due to the high number of degrees of freedom required to align 3D objects that lack signature elements, such as secondary structures. To find local structural similarities, 3D objects must undergo extensive rotational and translational transformations so that various local alignments may be sampled, and differences can be measured. Such transformations impose a large overhead for computational methods. Several algorithms were reported to overcome the computational complexity arising from the spatial degrees of freedom (Nussinov and Wolfson 1991; Lin and Nussinov 1996; Brakoulias and Jackson 2004; Shulman-Peleg et al. 2004; Morris et al. 2005; Gold and Jackson 2006; Wallace et al. 2008, Venkatraman et al. 2009; Yin et al. 2009; Kihara et al. 2011; Zhu et al. 2015; Budowski-Tal et al. 2018; Daberdaku and Ferrari 2019). However, these methods rely on human-crafted descriptors and parameters based on heuristics, which may not be optimal in capturing the full complexity of molecular surfaces. In contrast to sequence representation (e.g. a string of amino acids) and structure representations (e.g. graphs whose nodes are atoms or residues), molecular surface representations are comprised of features at a higher level of abstraction such as chemical and geometric properties. Recently, Molecular Surface Interaction Fingerprint (MaSIF; Gainza et al. 2020) was introduced, which uses a geometric deep learning approach to describe fixed-size surface patches based on the distribution of chemical and geometric features within the patches. MaSIF provided an effective computational method to efficiently predict protein–protein and protein–ligand interactions. However, MaSIF is an application-dependent method focusing on interaction prediction, which is not directly applicable to surface similarity comparison.

Here, we present a method named Surface ID, which numerically encodes protein surfaces, and efficiently assesses similarity with high accuracy. First, Surface ID follows the MaSIF preprocessing workflow(Gainza et al. 2020) to represent a molecule’s surface as a triangular mesh and precomputes chemical and geometric features for each vertex point on the mesh. Then, it defines a surface patch of a fixed geodesic radius at each vertex point and uses a geometric convolutional neural network to encode the surface patches into descriptors of a fixed dimension. The network is trained in a self-supervised contrastive manner (Chopra et al. 2005), in which surface patches with spatial proximity to each other on the same protein are forced to have similar encodings. By computing descriptor distance within the encoding space and optionally performing a clustering and alignment algorithm, the method identifies the surface regions that share similar properties. We illustrate Surface ID’s capability in protein–protein interaction (PPI) classification and antibody epitope and paratope clustering. Furthermore, we showcase an application of Surface ID in antibody discovery by searching an epitope database for similar surface patches to a given query molecule.

## 2 Materials and methods

Detailed methods can be found in Supplementary Material.

## 3 Results

### 3.1 Surface ID learns a meaningful representation of protein surfaces

**Figure 1.**
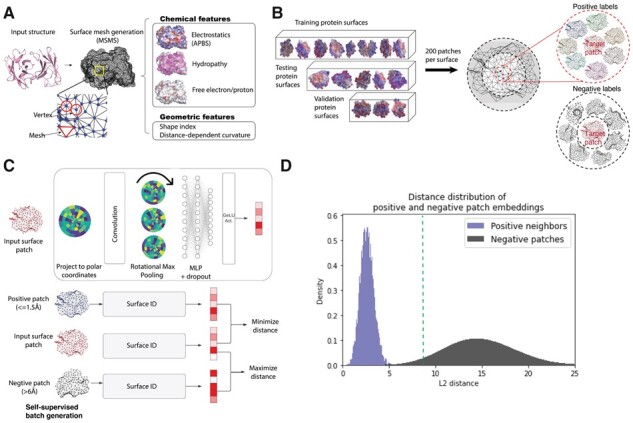
An illustration of preprocessing and self-supervised contrastive learning. (A) Upon protonation, the solvent-accessible surface for each protein was generated using the MSMS package. Subsequently, the protein surface was featurized with geometrical and chemical features. (B) The training, testing, and validation datasets were generated using 200 surface patches on each protein surface within each set comprising 2700 × 200, 100 × 200, and 50 × 200 surface patches for training, testing and validation, respectively. For each patch, positive/similar surface patches were generated from which were centered on the vertices located within 1.5 Å of the central vertex. Surface patches centered on vertices outside 5 Å cutoff were defined as negative/dissimilar surface patches. (C) Vertex points of a surface patch with their corresponding features were projected onto a local soft pixel grid, with 5 radial and 16 angular bins, using a Gaussian kernel with learnable parameters. A geometrical deep learning model was trained on the soft pixel grids to minimize the Euclidean embedding distance between neighboring patches and maximize the distance between distant, dissimilar patches. (D) The distribution of pairwise Euclidean embedding distance of each patch in the test set to nearby “positive” patches and “negative” patches. The green-dotted line represents a potential distance threshold for classifying embedding as functionally similar or different.

Surface ID uses self-supervised learning to learn patch embeddings. That is, during model training, a given patch embedding is trained to be similar to the embeddings of nearby patches on the same molecular structure and different from distant patches (Fig. 1A–C). Constraining nearby patches to have similar embeddings is natural as they likely compose together a larger surface region with a specific function. For example, if both patches are within the same local region of a binding interface, their embeddings should be similar. This idea extends beyond binding, however, to other functional properties such as aggregation propensity. To encourage a spatial resolution and prevent over-smoothing of embeddings over a large surface region, however, Surface ID trains the embeddings so that they are dissimilar for patches that are more than 5 Å apart. Moreover, during data preparation, the data was split by protein structures (Fig. 1B and Supplementary Material S1), as opposed to patches, to avoid data leakage and to evaluate Surface ID’s ability to generalize to entirely unseen structures.

Figure 1D illustrates the pairwise Euclidean distances between all the patch embeddings in the test set and each of their corresponding positive patches (<1.5 Å) and negative patches (>5 Å). As intended, the embedding of each patch of interest is more similar to nearby patches than distant patches. These distances form a bimodal distribution, suggesting a threshold to classify an unseen pair of patches as similar or dissimilar. Using the Euclidean distance as a proxy for the functional similarity between surface patches may be useful for many applications in protein engineering.

**Figure 2.**
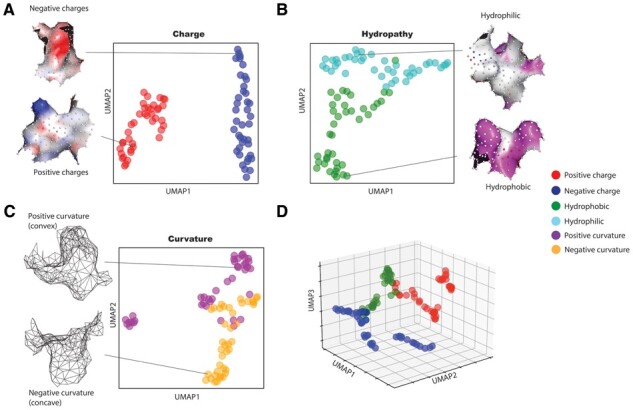
Surface ID learns a meaningful representation of protein surfaces. (A–C) 2D UMAP plots of the Surface ID embeddings from the selected surface patches (40 patches for each property, e.g. positive charge) showing Surface ID has learnt properties including electrostatic charges (A), hydropathy (B), and curvature (C). Representative surface patches for each property are shown. (D) 3D UMAP plot of the Surface ID embeddings from the selected surface patches showing the separation of positive charge, negative charge, and hydrophobic surface patches. The color scheme used in the UMAP plots for (A–D) is shown on the right.

To assess the functional similarity of embeddings, we compared their pairwise distances with charge, hydropathy, and curvature. Figure 2 shows low-dimensional Uniform Manifold Approximation (UMAP; McInnes et al. 2018) plots of the embeddings of 40 representative surface patches with extreme values for each of the functional properties. The UMAP parameters including n_neighbors, min_dist, metric, were set to 15, 0.1, and Euclidean, respectively. As shown, the embeddings are separated into clusters based on the chemical properties of charge and hydropathy as well as the geometric property of curvature, indicating that Surface ID captures surface properties. Even when patches on different proteins were compared, patches with similar embeddings had related chemical and geometric properties. In contrast, patches with a large embedding distance had very different properties.

### 3.2 Surface ID benchmarking

To demonstrate Surface ID’s utility, we benchmarked it in two different applications:

1. PPI classification.
2. Antibody epitope and paratope clustering.

The classification and clustering of the PPI systems based on the interface similarity can be utilized in a wide range of applications, such as functional annotation of proteins that lack evolutionary relatives and epitope/paratope clustering.

#### 3.2.1 PPI classification

**Figure 3.**
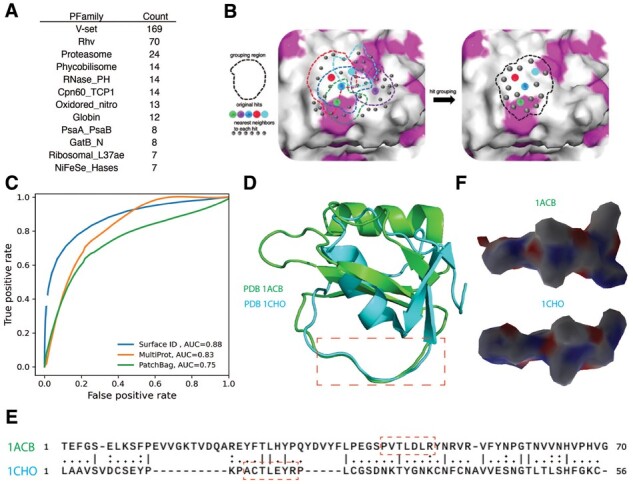
Performance of Surface ID in PPI classification. (A) PFAM protein families with PPI counts were used in this benchmarking. (B) An illustration of the grouping protocols used following the hit identification. The vertex points on each surface that fall within a defined distance are grouped together. In this illustration, the purple color vertex does not fall within the defined neighborhood of any other hit vertex and is excluded from the group. Next, the group is extended to the nearby neighbors, as defined using a geodesic distance cutoff, of each hit vertex in the group. Finally, the reciprocal mean descriptor distance is provided as a metric for surface area similarity. (C) The performance of Surface ID compared with MultiProt and PatchBag in PPI classification. (D–F) A representative example showing two proteins with low sequence and structure similarity possess similar surface patches. (D) Superimposition of PDB 1ACB and PDB 1CHO. Red dotted line box indicates a local similar loop (E) Sequence alignment showing low sequence similarity. The region superimposed in the red box region in (D) is not aligned correctly. (F) Electrostatic surfaces of the loops from 1ACB (top) and 1CHO (bottom) showing shape and charge similarities between them.

PPI classification of proteins with poor sequence similarity is a challenging task (Zhang and Kabuka 2021). We hypothesized that protein surface representation can be more informative for classifying PPI than sequence features. Here, we tested the capability of Surface ID in PPI classification to their corresponding protein domains (based on PFamily classes [Punta et al. 2012]) using a subset of the database from the 3DID server (Budowski-Tal et al. 2018). As described by the authors, upon clustering the 3DID systems based on their sequence similarity and using the cluster heads as group representative and eliminating small peptides (size<50), 1070 PPI systems were labeled with their corresponding PFamily groups (Fig. 3A). Using the definition of the interface residues in the dataset, surface patches within 3.5 Å from these residues were designated as the query region and fed into the Surface ID to compute descriptor distances for all pairs of the query and candidate surface patches. The candidate surface patches that are within the descriptor distance cutoff are grouped and extended as shown in Fig. 3B and described in Section 2. This serves three purposes: (i) it ensures that a substantial surface area on both query and candidate molecules are similar; (ii) the overlapping surface regions characterized as hit are joined to form a continuous surface that can be used to align the surfaces; and (iii) the interface surface is not limited by the 6 Å-radius, which is the minimum unit that Surface ID uses to perform convolution to learn its local geometric and chemical properties (described in Section 2).

Once the query and candidate groups are defined, to assess the interface similarity, we use the interface similarity score SS_AB_ between Proteins A and B as proposed by Budowski-Tal et al. (2018) which is the product of the similarity scores ss_AB_ and ss_A′B′_ between their binding partners as follows: where A′ and B′ are the proteins in complex with Proteins A and B, respectively, and the ss_AB_ and ss_A′B′_ are the reciprocal means of the descriptor distances between Proteins A and B and proteins A′ and B′, respectively. These joint SS_AB_ values were used to perform the ROC-area under the curve (AUC) analysis.


Eq. 1 $${\text{SS}}_{\text{AB}}={\;\text{ss}}_{\text{AB}}\times {\;\text{ss}}_{A\prime B\prime }$$

We benchmarked Surface ID against two different state-of-the-art approaches, PatchBag (Budowski-Tal et al. 2018) and MultiProt (Shatsky et al. 2004). Although not using machine learning (ML), PatchBag is a surface-based method for efficient comparison of protein surfaces and interfaces. It showed comparable performance to alignment-based structural comparison methods, such as MultiProt. As presented in Fig. 3C, the Surface ID obtained the ROC-AUC of 0.88, outperformed PatchBag (Budowski-Tal et al. 2018; ROC-AUC = 0.75) and MultiProt (Shatsky et al. 2004; ROC-AUC = 0.83).

To further demonstrate the advantages of Surface ID over sequence- or structure-based approaches, two proteins belong to chymotrypsin inhibitor family were compared. Although they share similar function of binding chymotrypsin, their overall structures and sequences are significantly different, e.g. sequence identity is <17% (Fig. 3D and E). Interestingly, Surface ID similarity scores (s1) of their surfaces around the chymotrypsin binding sites is 0.8, indicating high similarity. When examined in detail, we found the geometric and chemical properties around these surfaces are indeed similar, e.g. electrostatic properties shown in Fig. 3F. This shows the benefit of using local surface to search functional-related proteins, which cannot be easily done by sequence- and structure-based approaches.

#### 3.2.2 Epitope and paratope clustering

Instead of evolutionary conserved PPIs, antibody–antigen (Ab–Ag) interaction is driven by adaptive immune pressures, resulting in much higher diversity on the antibody side. For example, there can be multiple different antibodies binding to the same antigen, even on the same epitope. Therefore, antibody clustering requires higher sensitivity to subtle changes in paratope, while general PPI classification may classify antibodies into one big family. Moreover, in contrast to focusing on one side in the PPI classification, here we tested Surface ID to cluster paratope and epitope simultaneously, in order to compare the correspondence clusters between antibodies and antigens.

Traditionally, antibodies are clustered based on their clonotypes, especially on the heavy chain complementarity determining region 3 (HCDR3) diversity. However, such approaches fail to identify sequence-distant antibodies that target the same epitope. For instance, the CoV-AbDab server (Raybould et al. 2021) reported 4330 antibodies that bind to Severe acute respiratory syndrome coronavirus 2 ( SARS-CoV-2) antigens, 1785 of which target the receptor binding domain (RBD). Many sequence-diverse antibodies that belong to the same class bind to similar epitopes on RBD. This points to the need for an *in silico* clustering of antibodies using their functional paratopes. Given Surface ID’s capability of capturing functional surface fingerprints, we tested whether surface-based epitope and paratope clustering correlate with Ab–Ag paired recognitions.

**Figure 4.**
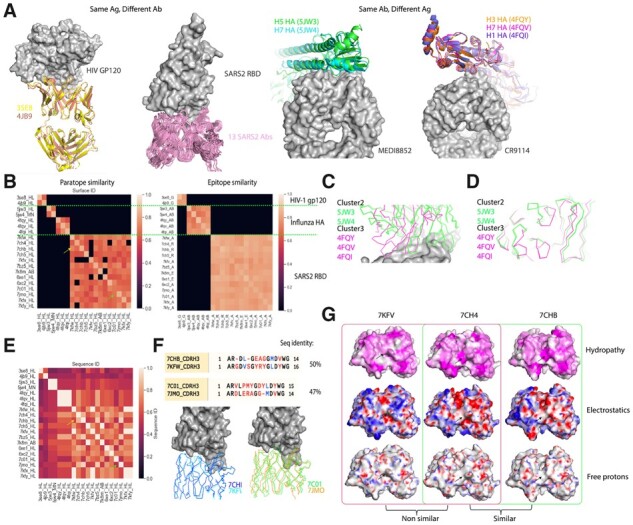
Epitope and paratope clustering based on the surface and sequence. (A) 20 Antibodies binding to the HIV-1 GP120, influenza HA, and RBD of SARS-CoV2 are used for clustering. The aligned structure of instances where similar antibodies bind to a different antigen or vice versa is illustrated in the panel. (B) Heatmap of the surface ID scores for paratope and epitope interface pairs, respectively. Two representative cases with high Surface ID similarity but low sequence identity are indicated by yellow and green arrows (the same as in E). (C) The two anti-HA antibodies bind to a similar epitope. (D) Paratope of the two anti-HA antibodies is different. (E) Heatmap of the sequence identities of HCDR3s from the 20 antibodies. (F) Four SARS-CoV-2 RBD antibodies with dissimilar HCDR3 sequences but similar surface scores. (G) Comparing paratope surfaces of 7KFV, 7CH4, and 7CHB, for nonsimilar and similar pairs on hydropathy, electrostatics, and free electron properties.

The dataset in this benchmark is comprised of 20 Ab–Ag complexes where the antigens belong to one of the three classes of HIV-1 GP120, influenza hemagglutinin (HA), and SARS-CoV-2 RBD with 2, 5, and 13 Ab–Ag complexes, respectively (Fig. 4A). For the HIV GP120 and RBD antigens, antibodies with different sequences bind to the same epitope. In contrast, for influenza HA, two broadly neutralizing antibodies bind to different HA antigens such as H1, H5, and H7 (Fig. 4A).

Following the IMGT (Giudicelli et al. 2006) annotation of the complementarity-determining regions (CDRs) using the ANARCI package (Dunbar and Deane 2016), the surface patches within 2 Å of the CDRs were used to define the paratope, whereas the antigen surface patches within 4.5 Å from antibody were used for epitope clustering. Subsequently, an all-against-all surface similarity search was carried out for paratope and epitope separately, using Surface Similarity Scores s1 as defined in Section 2 (Equation 1). Next, to cluster these interfaces, the ratio of similar surface vertices on the candidate interface (within descriptor distance of 3.5 from that of a target surface patch) to the total number of vertices on the interface was used as a similarity score. This ratio includes the expansion around the center vertex to include the nearby neighbor vertex points identified during the grouping step. Empirically, we found that the new metric helps capture small variations in the surface feature distribution and therefore provides a better metric for surface similarity.

As shown in Fig. 4B, Surface ID-based similarity comparison resulted in four clear paratope classes, corresponding to one cluster of antibodies for HIV-1 GP120, two for influenza HA and one for SARS-CoV-2 RBD. Although the two clusters of anti-HA antibodies target a similar epitope (Fig. 4C), the paratope structures are different (Fig. 4D). This explains the different clusters for the two anti-HA antibodies. Interestingly, there were three clusters for epitope, corresponding to three different antigens. Instead of two classes for the paratopes of the anti-HA antibodies, epitopes of all the five HA antigens from different strains (H1, H5, and H7) were clustered in one single class. This can be due to the extreme conservation of the stem region of HA across different strains, reflected by the fact that many broadly neutralizing antibodies target this specific region. Although overall surface features of the full HA antigens may not be similar, since Surface ID used a local surface patch for comparison, the epitope regions were shown to be similar. This highlights the benefit of using local surface features for functional classification.

To compare with sequence diversity-based clustering, sequence identities of the HCDR3 region (IMGT definition) from all the tested antibodies were plotted. Although the same normalization method was used, the contrast of sequence identity between similar and nonsimilar antibodies is much lower (Fig. 4E), indicating a finer resolution of Surface ID scores compared with sequence identity. Notably, HCDR3 from 7CHB and 7KFW showed relatively low sequence identity (50%); however, their surface similarity is high and comparable to those from other RBD antibody paratope pairs. This result was consistent with their binding specificity, as both bind to a very similar epitope in RBD (Fig. 4F). Another example of 7C01 and 7JMO was shown in Fig. 4F as well.

Similar to how the HA antibodies with different paratopes can bind to a similar epitope, three RBD antibodies, 7KFV, 7CH4, and 6XE1, showed paratope non-similarity to multiple RBD antibodies. For example, 7CH4 is similar to 7CHB, but not similar to 7KFV. We examined the comparison in detail by checking shapes and chemical properties of these surfaces. As shown in Fig. 4G, 7CHB and 7KFV shared similar features including hydropathy, electrostatics, and free hydrogen (e.g., areas indicated by the arrows), while 7KFV and 7CH4 differed significantly. Therefore, Surface ID similarity scores indeed reflected these surface similarities accurately. Despite a few pairs that may be flagged as nonsimilar by Surface ID, the paratopes of the 13 RBD antibodies largely showed in one cluster, suggesting a large number of redundant paratopes may help with robust clustering. Overall, these results demonstrated that Surface ID is able to unambiguously cluster antibodies based on their paratope surface diversity, which often relates to target specificity.

### 3.3 Application of surface ID in *in silico* antibody discovery

**Figure 5.**
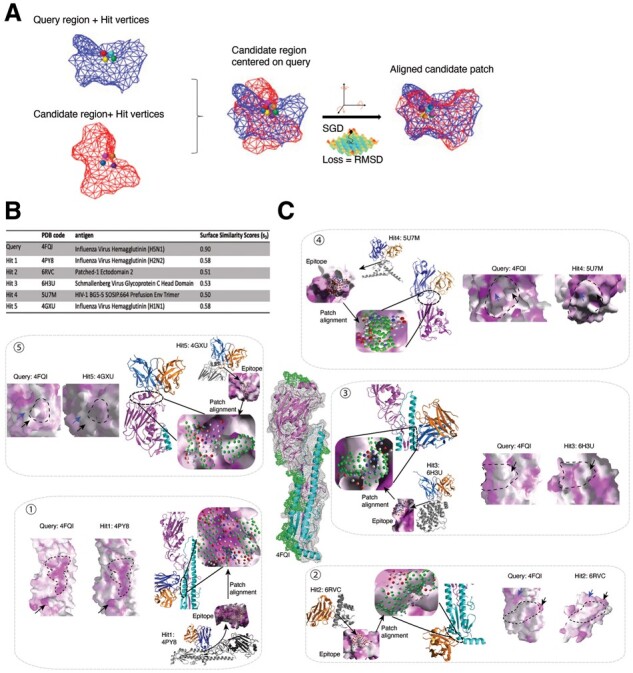
In silico antibody discovery by Surface ID. (A) Surface alignment process: starting from a target surface region and a candidate surface region and their corresponding matching vertices (left), candidate region is first centered on target by aligning the centroids (middle) followed by gradient descent rotational sampling to minimize loss, i.e. RMSD (right). (B) List of the five representative hits for the target of influenza virus HA (H5N1) the surface similarity scores (s3) in the table are calculated based on the Equation (3) described in Section 2. (C) Illustration of the five representative hits. The target (HA) is in the center with hit regions colored green. In each pop-up box: Fv structure of the antibody bound to the epitope in the library is colored blue (light chain) and orange (heavy chain); The target antigen (HA) and hit antigens are represented in cartoon and colored in magenta and gray. The target and hit vertices are colored in green and blue-red scale, respectively; The zoom in view shows target and hit alignment; After alignment, the grafted antibody bound to the target is shown as a complex structure; A side-by-side view of the epitope and the target regions are shown in surface representation, and colored based on hydropathy (hydrophobic in magenta and hydrophilic in white); Aligned areas are circled by a black-dashed line, and similar features are indicated by black and blue arrows.

Inspired by viruses hijacking host biological systems through functional mimicry of host proteins (Alcami, 2003), we sought to discover antibodies for a given antigen by searching similar epitopes from an existing epitope library, and grafting corresponding antibodies to the interested antigen as an engineering template. Since Surface ID showed a promising performance in surface similarity comparison, we tested its application in a *de novo* antibody discovery task. In this case, we took the HA protein from an H5N1 influenza virus (PDB code 4FQI) as the query antigen, and we searched all surface patches from the query against all 2850 epitopes in an epitope library, which was generated from a snapshot of the SAbDab database (7 November 2020; Dunbar et al. 2014). After a set of candidate surface regions potentially similar to a query surface region are found, each candidate region is geometrically aligned to the query region for a more granular evaluation of the pair’s similarity as described in Fig 5A and in Section 2.

In this search, 58 hits were obtained with reasonable Surface ID Scores (Fig. 5B and Section 2) across diverse regions of the target surface (Fig. 5C, center). Among them, 32 hits are HA antigens, which are expected given many HA antigens are within the epitope library (Supplementary Table S1). However, besides HA there were 26 unexpected non-HA hits, including glycoproteins from HIV-1 and Schmallenberg Virus, a hedgehog ligand receptor (PTCH1; Supplementary Table S2). In contrast, DALI search (Holm 2022) using the same query protein resulted from all HA antigens for the top 500 hits (Supplementary Material S2), demonstrating the benefit of using local surface-based search over global structural search for function site comparisons. When examining these surfaces in detail using a script we developed to automatically load query and hit surfaces, as well as the associated structures, we found the local surface regions of the hits and the targets are generally similar in shapes and chemical features (e.g. hydropathy; Fig. 5C, pop-up boxes). In the non-HA cases, no sequence or structure similarity can be identified between the query and hits. This demonstrates how Surface ID can provide an increased sensitivity for searching similar functional sites.

Since the surfaces in the library are all epitopes, antibodies associated with epitope hits can be grafted to similar surface patches of the target by the alignment algorithm. As shown in Fig. 5C (pop-up boxes), the grafted antibodies were properly placed in the different areas of the target. Given that Surface ID search is limited to a local region, depending on relative position of the target surface and its surrounding environment (e.g. spatial clashes or incompatible chemical properties), grafted antibodies may not be immediately applicable to binding the target. However, with the recent advancement in protein engineering and design (Adolf-Bryfogle et al. 2018; Cao et al. 2022; Jin et al. 2022), we expect using grafted antibodies as starting templates for downstream antibody optimization and design.

## 4 Discussion

In this work, we present a computational tool, Surface ID, as one of the first geometric deep learning models that are constructed to assess similarity between protein surface patches. Although it uses geodesic convolutions with Gaussian mixture soft pixels similar to MaSIF, Surface ID was implemented with several key innovations. First, Surface ID’s architecture was customized to obtain a meaningful representation of a surface patch that can be used for similarity comparison. Second, the Surface ID model was trained by a novel self-supervised strategy, where highly overlapping and random distal surface patches are considered as similar and dissimilar, respectively.

This allows leveraging the large amount of protein surfaces that can be generated from protein structure data and overcoming the labeled data limitation. In addition, Surface ID uses a novel grouping algorithm to extend the identified vertex hits. This step is critical as it avoids high false positive rate that can result from treating dissimilar surface patches as genuine hits due to low descriptor distances that can arise from statistical fluctuations. At the end, Surface ID outputs various metrics, including mean descriptor distance and the fraction of similar patches (defined by descriptor distance threshold) in identified clusters. This provides flexibility as different metrics may be used in different applications for optimal performance.

Compared with MaSIF that uses application-dependent neural networks, Surface ID generates descriptors that can be used directly for similarity comparison in various applications. This might be an important aspect that makes the Surface ID descriptor more robust and generalizable to different tasks as demonstrated in the benchmark tests. Moving one step further, Surface ID was tested for *in silico* antibody discovery by searching similar surfaces through a preconstructed epitope library. For this application, it is not the discovery of two similar *patches* of a fixed radius (e.g. 6 Å) centered on two different vertices that would be of interest, as is learned during training, but instead the discovery of a set of similar surface *regions* is sought. These regions could be as large as the entire surface of a protein for an exhaustive search as the “query” in the *in silico* discovery case, although the regions can be constrained to a particular boundary of interest (e.g. CDR3 of an antibody), number of vertices, and geodesic radius to be more efficient. Notably, given an epitope of interest, this method identifies the *largest* surface area that is similar by grouping a matching vertex with its nearby vertices. After hit identification and grouping, we implemented an alignment step to further compare the hit surfaces. This step is important for two major reasons: (i) It provides a second similarity score in addition to the one based on descriptor distance that further increases success rate by filtering out hits with low scores. (ii) It allows initial placement of the corresponding hit antibody by superimposition between query and hit surfaces. Moreover, we developed a python script that can automatically load query and hit surfaces after alignment, as well as the associated antigen and antibody structures, into PyMOL (Schrödinger LLC 2015) to allow direct visual examinations (Supplementary Material). This provides a starting template for downstream optimization/engineering, thereby enabling an *in silico* antibody discovery workflow.

Based on the benchmarking result and the promising hits identified in the *in silico* antibody discovery exercise, we believe Surface ID has learnt a meaningful representation of protein surfaces. Hence, we envision broader uses of Surface ID’s descriptors as a fingerprint embedding for further application-specific model development. In contrast to protein language model (PLM) embeddings which operate in residue or sequence level (Alley et al. 2019; Rives et al. 2021), the Surface ID embedding encodes chemical and geometric properties directly from 3D structure in a sequence-independent manner. Therefore, local features, which are related to function directly, can be enriched more efficiently. Compared with PLM embeddings, we expect Surface ID playing more important roles in functional characterization and engineering applications. Moreover, with the advent of highly accurate structure prediction models such as AlpahFold2 (Jumper et al. 2021) and RoseTTAFold (Baek et al. 2021), together with proteome-wide structure model databases (Varadi et al. 2022), Surface ID’s application can be extended to the whole proteome level for surface-based comparison or annotation. Finally, since Surface ID is based on molecular surfaces, it is agnostic to molecule types (e.g. DNA, RNA, and small molecule). Upon further system-specific training, Surface ID could be employed in nucleotide-related or small molecule-related systems, including DNA/RNA–protein interaction and small molecule drug discovery.

Although Surface ID exhibits promising performances on several fronts, it is prone to a few limitations, including not incorporating structural flexibility. One important factor in protein-protein interaction is the plasticity of the interface (Tuffery and Derreumaux 2012; Fernandez-Quintero et al. 2019), while the current implementation of Surface ID uses a static representation of the protein surface. This issue may be addressed in the future by the inclusion of dynamic features in the Surface ID workflow. Moreover, since the surface features rely on high-resolution information (e.g. side chain orientation), the accuracy of input structures highly impacts the result. For instance, the accuracy of the loop models is essential in predicting Ab–Ag clusters. This problem is expected to be alleviated with the progress in improving modeling accuracy. Finally, the computational cost of the preprocessing of protein structures can limit its applicability to perform the search on the large datasets, such as the entire PDB library. As suggested by Sverrisson et al. (2020), using point cloud representation of the protein surface and KeOps library (Charlier et al. 2021) for symbolic matrix manipulation can massively improve the performance of the Surface ID package.

## 5 Conclusions

With the advent of experimental methods, e.g. crystallography and cryogenic electron microscopy (cryo-EM), and the revolution in artificial intelligence techniques, structural libraries of protein systems are ever-growing. This offers a unique opportunity for computational methods that can search these libraries to identify a molecule with the desired characteristics. In this work, we demonstrate the applicability of Surface ID in PFamily classification of PPI systems and epitope–paratope clustering using the similarity of surface at the interface. The key strength of Surface ID is its generalizability to a wide range of molecular size and type. Furthermore, we highlighted the instances where Surface ID can be used for *de novo* antibody discovery. PPIs play pivotal roles in biological processes, and it’s a critical component for drug discovery, especially for biologics drugs. Despite the importance, PPI remains one of the most significant challenges. We believe fundamental methods like Surface ID are what will truly enable *de novo* design and virtual screening engines, and revolutionize drug discovery.

## Supplementary Material

btad196_Supplementary_Data

## Data Availability

The data underlying this article will be shared on reasonable request to the corresponding author.
