# Correction: Surface ID: a geometry-aware system for protein molecular surface comparison

**DOI:** 10.1093/bioinformatics/btad347

**Published:** 2023-06-05

This is a correction to: Saleh Riahi and others, Surface ID: a geometry-aware system for protein molecular surface comparison, *Bioinformatics*, Volume 39, Issue 4, April 2023, btad196, https://doi.org/10.1093/bioinformatics/btad196

In the originally published version of this manuscript, the 7th author was missed from the author list. This should read: “Saleh Riahi^1,*,†,^ Jae Hyeon Lee^2,5,†^, Taylor Sorenson^2^, Shuai Wei^1,6^, Sven Jager^3^, Reza Olfati-Saber^2^, Yanfeng Zhou^1,7^, Anna Park^1^, Maria Wendt^1^, Hervé Minoux^4,*^, Yu Qiu^1,*^” instead of: “Saleh Riahi^1,*,†,^ Jae Hyeon Lee^2,5,†^, Taylor Sorenson^2^, Shuai Wei^1,6^, Sven Jager^3^, Reza Olfati-Saber^2^, Anna Park^1^, Maria Wendt^1^, Hervé Minoux^4,*^, Yu Qiu^1,*^”.

This error has been emended in the article.

