# Supplementary material for: Surface ID: a geometry-aware system for protein molecular surface comparison
Source: Bioinformatics. 2023 Apr 17;39(4):btad196. doi: 10.1093/bioinformatics/btad196 (PMC10133531; doi:10.1093/bioinformatics/btad196)
Supplement: btad196_Supplementary_Data [file btad196_supplementary_data.zip › SUPPLEMENTAL_MATERIALS_final.docx]

**SUPPLEMENTAL MATERIALS**

**Surface ID: A Geometry-aware System for Protein Molecular Surface Comparison**

Saleh Riahi^1&^*, Jae Hyeon Lee^2&#^, Taylor Sorenson^2^, Shuai Wei^1†^, Sven Jager^3^, Reza Olfati-Saber^2^, Anna Park^1^, Maria Wendt^1^, Hervé Minoux^4^*, Yu Qiu^1^*

^1^ Large Molecule Research, Sanofi, 350 Water St, Cambridge, MA 02141

^2^ Data & Data Science, Sanofi, 450 Water Street, Cambridge, MA 02141

^3^ R&D Digital Data & Computational Sciences, Sanofi, Industriepark Hoechst,

Brüningstraße 50, 65929 Frankfurt am Main

^4^ Data & Data Science, Sanofi, 1 Avenue Pierre Brossolette, 91380 Chilly-Mazarin, France.

^&^These authors contributed equally.

*Correspondence to: Yu.Qiu@sanofi.com, Herve.Minoux@sanofi.com, or Saleh.Riahi@sanofi.com

^#^Current address: Prescient Design, Genentech, Inc., South San Francisco, CA 94080, USA

^†^ Current address: Bristol Myers Squibb, 100 Binney St, Cambridge, MA 02142

**Methods**

**Terminology and Definitions:**

- ***Surface***: Here we refer to the surface as the solvent accessible/exposed surface. It is the surface characterized around a protein by the solvent accessible area of the surface formed by hypothetical spheres centered at atom positions with the van der Waals radii.
- ***Mesh and vertex***: Protein solvent accessible/exposed surface is represented by a triangular mesh, which comprises a set of triangles in three dimensions that are connected by their common edges and vertices as shown in Figure 1A. Nodes of each triangle are referred to as “vertices”. A surface mesh of protein is made of thousands vertices.
- ***Surface patch***: As described in MaSIF, given a triangular mesh representation of a molecular surface, a surface patch defined at each vertex point is a set of vertices within a geodesic radius. In other words, each patch is built around a central vertex point. Therefore, for every vertex point on the protein surface, there can be a surface patch constructed around it. It is expected that surface patches originated from very close vertex points are highly overlapped. “Surface patch” and “vertex” are sometimes interchangeably used in the following text.
- ***Surface region***: A set of surface patches where each center vertex is within some geodesic distance of that of another vertex. In contrast to a surface patch, a surface region does not have a fixed radius.

**Preprocessing and Patch Featurization**

The surface triangulation and featurization implemented in MaSIF(Gainza, et al., 2020) was used to generate the surface patches (Figure 1A). Briefly, each protein is protonated using the Reduce package and the solvent accessible/exposed surface area is obtained by the MSMS program(Sanner, et al., 1996). Subsequently, the chemical features including hydropathy, free electrons/protons, and electrostatic charges are assigned using the hydropathy scale(Kyte and Doolittle, 1982), hydrogen bond potential(Kortemme, et al., 2003), and APBS’s Poisson-Boltzmann solver(Baker, et al., 2001), respectively. Geometric features (i.e., shape index) are computed based on principal curvatures(Koenderink and van Doorn, 1992). Distance dependent curvature is defined as the change in geodesic distance from patch center upon moving surface points along the local surface normals(Yin, et al., 2009). These featurized surface patches are input into the Surface ID algorithm to perform surface similarity searches.

**Learning a Latent Space Representation of the Protein Surface: Self-supervised Contrastive Learning**

Upon surface patch generation and featurization, we trained a geometric convolutional neural network whose architecture is similar to that of the neural network used in MaSIF-Search but with several modifications. We used 6 Å radius patches as input to the network with 5 radial bins and 16 angular bins for the soft pixel grid, with an additional center soft pixel. A single layer of convolution is performed in a depth-wise separable manner and is followed by rotational max pooling as in the MaSIF-Search module, with 5120 output channels. To produce the final output descriptor, the outputs of this network are input into a multi-layer perceptron network consisting of a hidden layer with 1024 nodes, GeLU activation, a dropout rate of 0.1, and hidden layer dimensionality of 1024.

To train the network, we used the triplet loss minimization strategy(Chechik, et al., 2010), though instead of minimizing the descriptor distance of interacting pairs of surface patches and maximizing the distance of non-interacting pairs as in MaSIF-search, the Euclidean L2 distance of similar patches was minimized, and the distance of dissimilar patches was maximized. Notably, the training data was generated in a self-supervised fashion. That is, during each mini-batch step of the training, a single vertex point of a pre-processed protein surface mesh is chosen at random. Within the 6 Å radius patch associated with the chosen vertex, all pairs of vertices that are within 1.5 Å in geodesic distance of each other are considered “positive” pairs and those outside 5.0 Å “negative” pairs with all other pairs being excluded (Figure 1B-C).

The Adam optimizer(Kingma and Ba, 2014) was used with default parameters, a learning rate of 5 × 10^-4^, and decoupled weight decay with penalty of 1 × 10^-2^. A manual, heuristic hyper-parameter search showed that the large number of output channels and long training beyond the initial flattening of the validation loss curve were important for performance. Other hyper-parameters such as soft pixel grid bin size, number of convolutional layers, and dense vs. separable convolutions were manually varied to find optimal parameters based on the validation set. For instance, networks with more than one convolutional layer required a smaller number of bins in the soft pixel grid due to the GPU memory constraint and did not perform better than the single layer network.

A snapshot of the SAbDab database (2020.11.07)(Dunbar, et al., 2014) was used to train the model. This dataset consists of 3,206 Ab/Ag pairs. Following the preprocessing 2,850 proteins were selected for the training of the model where the data was split into 2700, 100, and 50 for training, testing, and validation, respectively. For every molecule in the training, testing, and validation sets, 200 vertices were randomly selected, and the corresponding positive and negative patches were generated.

**Initial Hit Identification**

Given a query surface region composed of a set of vertices V_Q_, we have devised the following workflow to identify matching surface regions in a library of protein structures. The workflow first scans the library at the surface patch level by comparing neural network descriptors to find candidate surface regions that contain surface patches that are similar to those in the query region. Next, the hit vertices are grouped into candidate surface regions and scored against the query region using alignment-based methods. To perform the first step, all vertices in V_Q_ are compared with all vertices on the l-th candidate surface V_C_^l^ by calculating the Euclidean distance of their vertex descriptors. A vertex v_c_^j^ in V_C_^l^ with a descriptor distance less than a user defined threshold T to any vertex v_q_^i^ in V_Q_ is considered a hit vertex. That is, a candidate vertex v_c_^j^ in V_C_^l^ is a hit if:

d( F(v_q_^i^ ), F(v_c_^j^) ) ≤ T for any v_q_^i^ in V_Q_

where d is the Euclidean L2 distance and F is the embedding network described previously.

**Grouping Hit Surface Patches into Candidate Surface Regions**

Hit vertices that are within a Euclidean 3D distance cutoff, e.g., 3 Å, from one another are grouped together. Subsequently, groups that share at least one vertex are joined together and their neighboring vertices that are located within a distance cutoff, e.g., 2 Å, are added to the group**.** At this stage, any pairs of query and library surface regions that do not meet a user-chosen minimum number of hit vertices threshold (e.g., 40) are excluded from further consideration.

**Alignment of Candidate Surface Regions to Query Surface Region:**

Alignment is done by stochastic gradient descent (SGD) with the objective of minimizing the root-mean-square deviation (RMSD) between all possible vertex pairs of candidate and query by rotating and translating the candidate surface region. The SGD algorithm was used as it doesn not require correspondence between vertex points from query and hit surface patches, so that it serves as an orthogonal metric assessing surface similarity. Furthermore, as our alignment is implemented in PyTorch, it can utilize GPU parallelization with high efficiency. To initialize the alignment, the geometric centroids of the query and candidate regions are subtracted from the positions of their corresponding vertices, respectively. The resulting translated candidate region vertices are rotated with 3D rotations around X, Y, and Z uniformly sampled with a user defined interval, e.g., 20°. For each sampled rotation, the SGD proceeds until either the maximum number of alignment steps or the objective achieves a minimum threshold. The aligned pair with the minimum objective is selected and used for evaluating the overall similarity of the two regions. For example, to quantify the degree of surface similarity over an extend region after the SGD alignment, Surface ID Scores (SS) are defined and computed as the geometric mean of the ratios of the numbers of hit vertices to the total number of vertices in the hit regions for both candidate and query. This score can be used as a metric to characterize the similarity of query and candidate surface regions and rank the candidate regions obtained during an entire library search. The alignment method can be used to generate preliminary poses of hit antibodies on a query target surface.

**Surface Similarity Scores from Surface ID**

Following the hit identification with Surface ID several metrics/scores are computed that be used to characterize the extend of the surface similarity. Three similarity scores are computed before alignment and one is provided after the alignment step.

**Pre-alignment scores:**

1. **Fraction hits for target**

$\boldsymbol{s}_{\boldsymbol{1}}\boldsymbol{=}\frac{\boldsymbol{N}_{\boldsymbol{query}}^{\boldsymbol{hits}}}{\boldsymbol{N}_{\boldsymbol{extended query}}^{\boldsymbol{v}}}$ **Equation** (1)

Where the numerator is the number of the vertices of the target that satisfy the descriptor distance threshold. The denominator is the number of vertices on the target that are within the extended area as obtained by the grouping scheme.

1. $\boldsymbol{s}_{\boldsymbol{2}}\boldsymbol{=}\frac{\boldsymbol{N}_{\boldsymbol{candidate}}^{\boldsymbol{hits}}}{\boldsymbol{N}_{\boldsymbol{extended candidate}}^{\boldsymbol{v}}}$ ***Equation (2)***

same as s_1_, but the vertices belong to the candidate surface area.

1. $\boldsymbol{s}_{\boldsymbol{3}}\boldsymbol{=}\sqrt{\boldsymbol{s}_{\boldsymbol{1}}\boldsymbol{s}_{\boldsymbol{2}}}$ ***Equation (3)***
2. $s_{4}=\#\left\{ r_{q,c} \right| QC : r_{q,c}<1.5 \}$ ***Equation (4)***

Number of vertices on query and candidate that are within 1.5 Å from each other following the alignment.

**Table S1. List of 32 HA candidate hit surface regions and their Surface ID Scores (SS) using 4FQI antigen as the query protein surface.**

| PDB ID | antigen | S_3_ |
| --- | --- | --- |
| 6P3R | influenza A H5N1 Vietnam hemagglutinin head | 0.721 |
| 6A0Z | H5N1 influenza hemagglutinin, HA head region | 0.689 |
| 3GBM | H5N1 influenza virus hemagglutinin | 0.672 |
| 4K3J | influenza h5 ha head domain vietnam | 0.645 |
| 4XRC | hemagglutinin 5 (H5) | 0.643 |
| 6A67 | influenza A virus H5 hemagglutinin globular head | 0.639 |
| 6IUV | influenza A virus H5 hemagglutinin globular | 0.636 |
| 4XNM | hemagglutinin 5 (H5) | 0.632 |
| 1EO8 | influenza virus hemagglutin (ha1 chain) | 0.625 |
| 6IUT | influenza A virus H5 hemagglutinin globular head | 0.612 |
| 6OC3 | H1 (A/Solomon Islands/3/2006) | 0.610 |
| 5UGY | H1N1 strain A/Solomon Islands/3/2006 HA | 0.610 |
| 3LZF | 1918 influenza hemagglutinin | 0.607 |
| 6Q0H | hemagglutinin head A/Beijing/262/95(H1N1) | 0.605 |
| 5GJS | H1 hemagglutinin from A/California/04/2009 | 0.594 |
| 6E3H | H5 influenza hemagglutinin | 0.593 |
| 4R8W | H7 hemagglutinin from A/Anhui/1/2013 | 0.592 |
| 5XHV | Influenza Hemagglutinin, HA1 subunit | 0.589 |
| 6A4K | H1N1 influenza A virus HA1 | 0.580 |
| 4PY8 | 1918 influenza virus hemagglutinin | 0.577 |
| 4GXU | 1918 influenza hemagglutinin | 0.576 |
| 4KVN | hemagglutinin A/Perth/16/2009 (H3N2) | 0.561 |
| 3SM5 | H1N1 strain A/Solomon Islands/3/2006 | 0.560 |
| 5DUP | influenza A H5 hemagglutinin globular head | 0.557 |
| 4M5Z | 2009 influenza hemagglutinin, HA1 subunit | 0.552 |
| 3GBN | 1918 H1N1 influenza virus hemagglutinin | 0.551 |
| 3SDY | influenza A H3 Hemagglutinin | 0.545 |
| 4HG4 | H2N2 influenza virus hemagglutinin | 0.543 |
| 5K9K | Influenza A Hemagglutinin (Hong Kong 1968) | 0.529 |
| 4YJZ | hemagglutinin H1 Solomon Islands/03/2006 | 0.521 |
| 6FYT | H1 (A/solomon Islands/3/06) Hemagglutinin | 0.515 |
| 4FP8 | H3 influenza hemagglutinin, HA1 subunit | 0.509 |

**Table S2. List of 26 non-HA candidate hit surface regions and their Surface ID Scores (SS) using 4FQI antigen as the query protein surface.**

| PDB ID | Antigen | S_3_ |
| --- | --- | --- |
| 6CMG | glycoprotein g | 0.678 |
| 2R56 | beta-lactoglobulin | 0.671 |
| 6LI3 | guanine nucleotide-binding protein | 0.659 |
| 3NFP | interleukin-2 receptor subunit alpha | 0.659 |
| 6CBV | BRILL | 0.656 |
| 4RWY | HIV-1 yu2 gp120 | 0.652 |
| 3K7U | MP18 RNA editing complex protein | 0.648 |
| 4K3J | hepatocyte growth factor beta chain | 0.645 |
| 3UC0 | envelope protein ectodomain dengue virus | 0.639 |
| 3VI4 | integrin beta-1 | 0.638 |
| 5E7F | major structural protein 1 | 0.634 |
| 5VXM | IpaD from Shigella flexneri | 0.630 |
| 1BGX | Taq DNA polymerase | 0.629 |
| 4LST | envelope glycoprotein gp120 of HIV-1 clade | 0.627 |
| 6HJY | cys-loop ligand-gated ion channel | 0.626 |
| 6E63 | malaria antigen Pfs48/45 | 0.624 |
| 6HHU | Bacillus anthracis Sap | 0.622 |
| 6HJX | cys-loop ligand-gated ion channel | 0.619 |
| 5UDC | fusion glycoprotein f0 | 0.618 |
| 4XNZ | envelope glycoprotein gp160 | 0.616 |
| 5JA8 | HigB2 toxin | 0.615 |
| 6M58 | serum albumin | 0.615 |
| 6GKU | galectin-10 | 0.614 |
| 6H3U | Schmallenberg virus Gc head domain | 0.533 |
| 6RVC | Patched-1 ectodomain 2 | 0.508 |
| 5U7M | HIV-1 BG505 SOSIP.664 Perfusion Trimer | 0.497 |

**Visualization of the hits surface patches**

The distributed PyMOL script built upon the MaSIF’s PyMOL plugin can be used to visualize the identified hits from a Surface ID search.

The following instructions can be used to load the library and query surface patch hits into the Pymol session for inspection and visualization purposes:

1. Run Surface ID with SAVE_PLY=TRUE

1. Copy the Surface ID’s PyMOL plugin into your work directory

1. Import the Surface ID plugin function into your load_hits.py script. An example of the load_hits.py script is provided below:

from pymol import cmd

sys.path.append('path_to_workdir')

from surface_id_plugin import *

hits_directory = "path_to_hits_directory"

hits = ['6rvc_B']

cmd.load('4fqi.pdb','4fqi')

load_ply(f"{hits_directory}/4fqi_AB_ref.ply", dotSize=0.5)

for hit in hits:

load_ply(f"{hits_directory}/{hit}.ply",dotSize=0.5)

Upon running the load_hits.py script with PyMOL, the aligned candidate hit and query surface patches are loaded and can be inspected as presented in Figure S1.


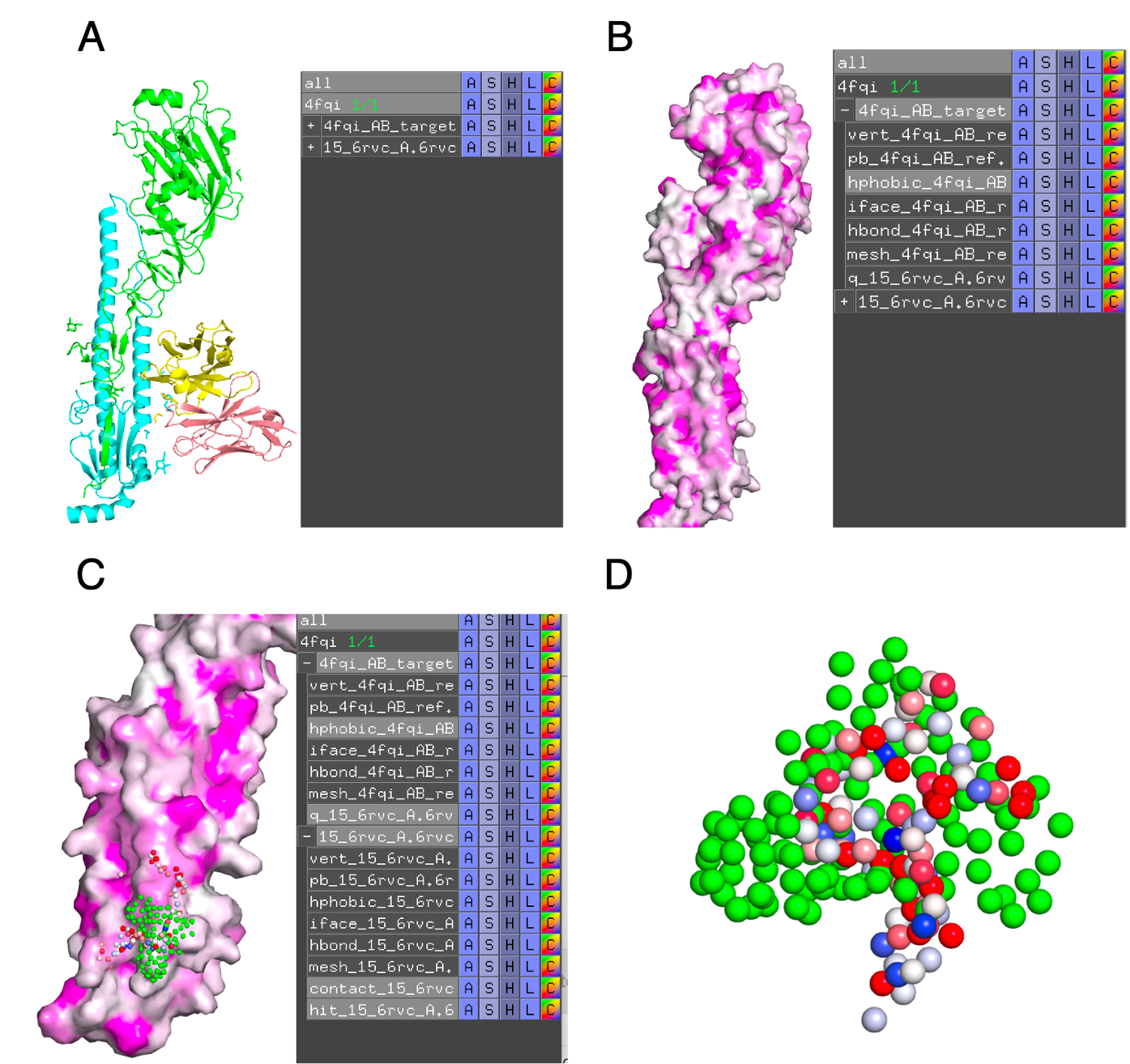


**Figure S1. Snapshots of the PyMOL window used for the visualization of candidate and query (4FQI antigen) surface patch hits.** **(A-B)** The query antigen structure and surface used during the search with a hit surface patch loaded upon alignment. **(C)** The hit region on 4FQI and 6RVC antigens. **(D)** An alignment of the corresponding hit regions between candidate and query where the query vertices are colored in green.

**Code availability**

Source code for the Surface ID model, trained weights and inference script are available under an open-source (Apache Version 2.0) license at <https://github.com/Sanofi-Public/LMR-SurfaceID>. In addition, scripts for visualizing automatically load query and hit surfaces after alignment

Data analysis used Python v.3.7 (https://www.python.org/), NumPy v.1.16.4 (https://github.com/numpy/numpy), SciPy v.1.2.1 (<https://www.scipy.org/>), Matplotlib v.3.3.4 (<https://github.com/matplotlib/matplotlib>) Neural networks were developed with pytorch (v 1.12).

Structure visualizations were created in Pymol v.2.3.0 (<https://github.com/schrodinger/pymol-open-source>).
